# Availability and outcomes of radiotherapy in Central Poland during the 2005-2012 period - an observational study

**DOI:** 10.1186/s12885-015-1236-7

**Published:** 2015-04-02

**Authors:** Justyna Chalubinska-Fendler, Wojciech Fendler, Michal Spych, Jolanta Luniewska-Bury, Wojciech Mlynarski, Jacek Fijuth

**Affiliations:** 1 Department of Radiotherapy, Medical University of Lodz, 4 Paderewskiego Street, 93-509 Lodz, Poland; 2 Department of Paediatrics, Oncology, Haematology and Diabetology, Medical University of Lodz, Lodz, Poland

## Abstract

**Background:**

Using a cross-database integrative approach, we performed an epidemiological analysis in a representative region of central Poland to evaluate the availability of radiotherapy (RTx) and overall survival of adult patients undergoing RTx for cancer.

**Methods:**

Epidemiological data on cancer incidence in the 2005–2012 period were obtained from the Nationwide Cancer Registry. Using data from the Ministry of Internal Affairs, we collected survival information of all patients treated in the only centre providing RTx for a region inhabited by approximately 2.6 million people.

**Results:**

After filtering out individuals on the basis of exclusion criteria, the final dataset covered 17,736 patients. Availability of RTx increased marginally, from 23.5% (2005) to 24.4% (2011, R = 0.39, p = 0.38), with the highest values noted in patients with cervical (78.5%), prostate (70.6%) and breast cancer (62.7%). However, due to the decreasing population of the region, we noted increasing disparity in the likelihood of receiving RTx depending on the patient’s area of residence, with rural areas becoming progressively more neglected. The best prognosis was noted among patients with breast or prostate cancer with 5-year OS rates reaching 81.2% and 83.3%, respectively. Multivariate analysis controlling for type of diagnosis and patient age showed a time-dependent improvement in outcomes (HR(95% CI): 0.96(0.94-0.98); p < 0.0001).

**Conclusions:**

Availability of RTx in Poland is still below that reported by developed European centres. Survival of patients undergoing radical RTx has gradually improved, although it is still below that of leading RTx departments, potentially due to delayed diagnosis or organisational barriers, necessitating further investigations.

**Electronic supplementary material:**

The online version of this article (doi:10.1186/s12885-015-1236-7) contains supplementary material, which is available to authorized users.

## Background

Population-based survival studies are a cornerstone of assessments of healthcare system efficacy [1]. The EUROCARE network collects survival data of patients with malignancies from European countries, thus allowing unbiased comparisons amongst countries and continents [2]. However, despite recent advances in computer use in clinical practice, Poland is still struggling with digitization of its healthcare registries, distribution of radiotherapeutic equipment and staff limiting the scope of epidemiological analyses of the Polish population [3]. This makes it extremely difficult to perform comparative analyses of prevalence, whilst estimation of survival is nearly impossible. However, an opportunity to perform such analyses arose owing to the highly centralised network of radiation oncology departments which exists in the publicly funded oncological treatment system of Poland. Within the 16 administrative regions of Poland (voivodeships) there are only 23 radiation oncology centres, which thus constitute foci for epidemiological analyses in oncology. In the Lodzkie Voivodeship, a single, large centre oversees radiotherapy (RTx) for all eligible patients with cancer, and has been running a computerised medical database since January 2005. We have used this resource to demonstrate the development of a framework for nationwide integrative database construction and present the methodology and efficiency of a cross-registry search, focused on evaluation of RTx availability and survival of cancer patients.

## Methods

The study aimed to synthesise oncological data from regional and national levels, integrate it with place of residence information and provide an epidemiological reference on radiotherapy accessibility and survivorship in the Lodzkie Voivodeship. Epidemiological data on the number of newly diagnosed cancer cases in the Lodzkie Region were obtained from the nationwide cancer registry (data available for 2005–2012). This dataset is collated using reports from oncology centres and financial data of the National Health Fund. The registry is overseen by the National Centre of Oncology (NCO). The NCO dataset was previously used for epidemiological reports [4,5], and we used it here as the reference for evaluation of RTx availability.

To analyse RTx-related data collected at department level, we used the computer-based dataset of all patients treated in the Department of Radiotherapy of the Medical University of Lodz During the study period, this department was the only specialist radiation oncology centre for the Lodzkie Voivodeship, a region inhabited by 2.53-2.58 million people according to Central Statistical Office data (http://www.stat.gov.pl/bdlen/app/strona.html?p_name=indeks). The department’s database was installed in October 2004 and became the principal data storage resource for the department in January 2005. The database was constructed using Microsoft Access architecture (Microsoft, Redmond, WA, USA). It is continuously updated on a daily basis and manually curated. Data entry is performed by a team of three dedicated medical secretaries, and correctness of the medical records is verified by each patient’s attending doctor before the patient’s discharge. Data on diagnosis is coded using the ICD-10 classification as legally required by the National Health Fund. Since no long-term malfunctions of the database occurred during the analysed period of 1 January 2005–14 July 2012, we assumed that the database covers 100% of patients undergoing RTx. The number of patients from urban and rural areas, treated per year, procedures performed within the centre during that period, and times of introduction of novel techniques and equipment upgrades are shown in Additional file 1: Figure S1. Rural residents were individuals who inhabited rural areas or towns with a population density lower than 150 individuals/km^2^ as designated by the Central Statistical Office.

Survival data were obtained by integrating the department’s dataset with the nationwide, centrally-curated administrative PESEL database. The PESEL is the personal identification number assigned to each Polish citizen and used for most administrative purposes, including the healthcare system. Individual survival data are overseen by the Ministry of Internal Affairs. The repository is accessible to public medical institutions and we were thus able to obtain individual data confirming whether the designated patients were alive or dead at the time of database query (14 July 2012). This date constituted the final observation date – patients alive on that day were thus considered as censored observations. As the PESEL database is the main source of information on individual survival, we surmised that it can be used for survival analysis purposes without further validation. The latency of administrative data collection is no more than 14 days. Where possible, we cross-matched the administrative records with data on earlier studies performed in the centre [6-8], obtaining 100% concordance of survival data.

Patients could be referred to the department from all hospitals or clinics in the region following initial surgery or chemotherapy. Because the exact date of cancer diagnosis by histopathological examination was not available for the whole dataset due to disseminated data storage, the date of RTx initiation was established as the observation start for the analyses covered herein.

In order to make the analysis uniform, we excluded individuals who: were under 18 years of age at the time of RTx initiation; had undergone an earlier course of RTx; had received RTx for diseases other than cancer; had incorrect, incomplete or missing data on the date of RTx start or were impossible to match with the PESEL database.

The department’s database was expanded to cover demographic data on each patient’s address as well as information on total radiation dose and fractionation regimen, allowing for future in-depth clinical analyses. The patients were further divided into ones who received radical treatment, (intention-to-treat radiotherapy) and ones that received palliative treatment (for symptom relief). During the period covered by the analysis, several patients received concurrent chemotherapy in accordance to tumour-specific guidelines and therapeutic protocols depending on the attending oncologist’s discretion. Concurrent chemoradiation was given for cervical cancer, rectal cancer, head and neck cancers. In our centre lung cancer patients were treated with sequential chemoradiation throughout the analysed period. Small cell lung cancer patients after sequential chemoradiation with at least stabilization of a disease were referred for PCI (prophylactic cranial irradiation).

All patients agreed for the use of their clinical data in epidemiological studies by signing informed consent forms prior to the initiation of radiotherapy. Bioethics Committee of the Medical University of Lodz approved the study’s design.

### Statistical analysis

Continuous variables are given as medians with quartile boundaries (25-75%). Survival analysis was performed using the log-rank test and Cox proportional hazard regression. Hazard ratios (HR) with 95% Confidence Intervals (95% CI) were computed where possible. Trend analysis was performed using Pearson’s correlation coefficient. A p value <0.05 was considered as statistically significant. Statistica 10.0 PL software was used for statistical analysis (StatSoft, Tulsa, OK, USA).

## Results

**Figure 1:**
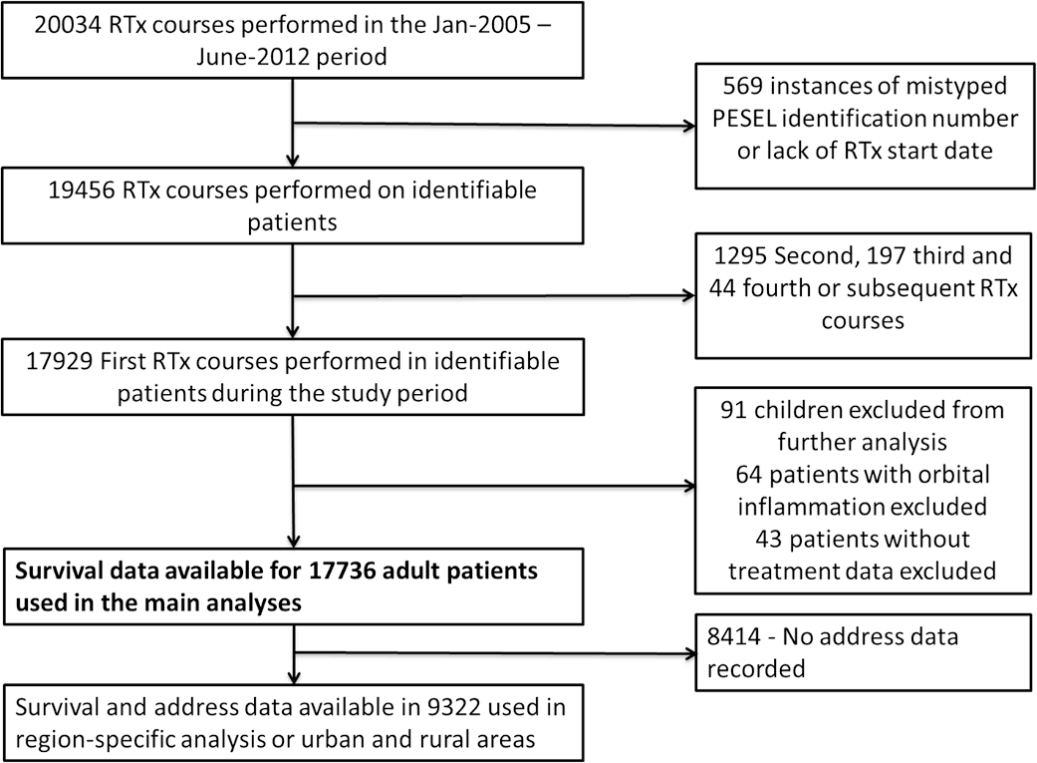
**Flowchart of data collection and patient selection.** The dataset marked in bold was used in primary outcome analyses. Individuals with data on place of residency were entered into region-specific analyses. RTx – radiotherapy; PESEL – unique personal identification number used in Poland.

**Figure 2:**
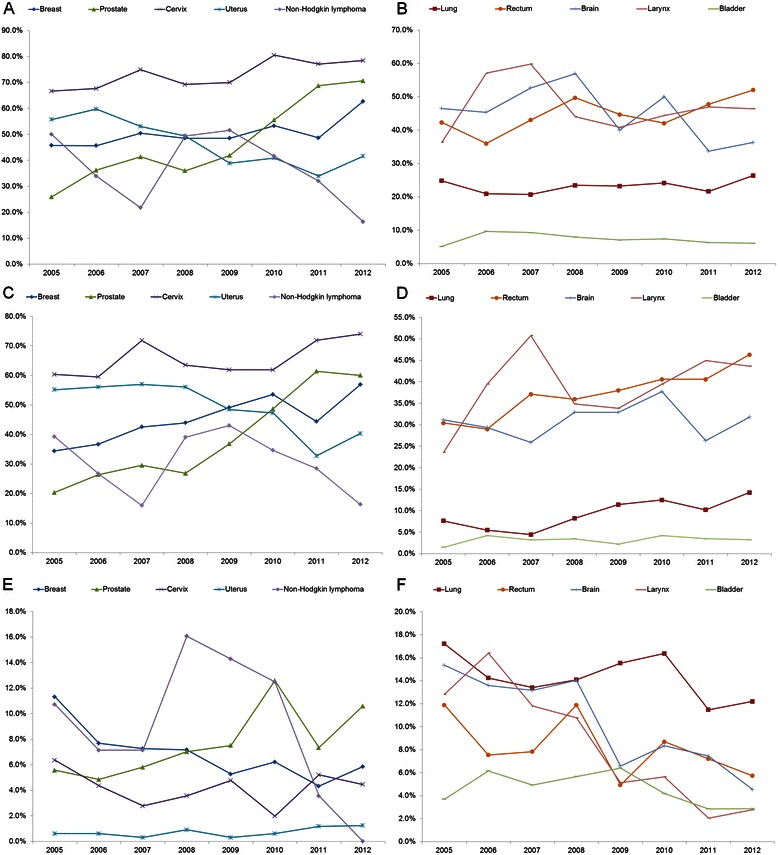
**The percentage of patients with specific diagnoses in the region who underwent treatment in the Center throughout the study period.** Percentage of patients with most frequent diagnoses with good **(A)** or poor **(B)** prognosis who underwent radiotherapy during the study period. Percentage of individuals undergoing RTx depending on diagnosis of the ten most frequently diagnosed types of cancer with good and poor prognosis divided into subgroups of radical **(C/D)** and palliative **(E/F)** RTx expressed as percentages of all patients diagnosed with cancer in the Lodz voivodeship.

The department’s dataset covered originally 20,034 patients. After filtering out individuals on the basis of exclusion criteria, the final dataset comprised 17,736 patients for survival analysis. A flowchart showing dataset development and the numbers of available individuals and reasons for exclusion are presented in Figure 1. The numbers of patients that began RTx in each of the analysed years, their exact diagnoses, and type of treatment used are shown in Table 1. The percentage distributions of the 10 most frequent diagnoses are presented in Additional file 1: Figure S2 (2A for radical RTx and 2B for palliative RTx). Throughout the analysis period, the percentage of availability by RTx of cancer patients increased marginally, from 23.52% in 2005 of newly diagnosed cancer cases to 24.41% in 2011 (R = 0.39, p = 0.38; Additional file 1: Table S1). The median age of the studied patients was 60.37 (25-75% 53.34-68.12) years, and 58.41% (N = 10,359) were female and 41.59% (N = 7,377) were male. The age of patients entering RTx correlated linearly with the year of study (r = 0.95; p < 0.001), with a mean increase of 0.41 years per year, which was statistically significant amongst breast, lung, prostate, rectal and laryngeal cancer (Additional file 1: Table S2). However, we noted an increase in the proportion of patients entering radical RTx (R = 0.89; p = 0.0068), which was paired with a proportional decrease in the proportion of patients undergoing palliative RTx (R = −0.86; p = 0.0137). Overall, more than 50% of individuals with cancer of the breast, prostate, rectum or cervix underwent RTx during the analysis period (Figure 2A/B). The percentage of newly diagnosed cancer patients entering radical RTx was positively correlated with the year of diagnosis in the case of breast (R = 0.89; p = 0.0068), lung (R = 0.75; p = 0.05), prostate (R = 0.96; p = 0.0005) and rectal cancer (R = 0.89; p = 0.0068; Figure 2C/D). No significant trends were noted for cancer of the uterus, cervix, brain, larynx, bladder or non-Hodgkin lymphoma. The decrease in the percentage of patients undergoing palliative RTx was statistically significant for breast (R = −0.96; p = 0.0005), brain tumours (R = −0.79; p = 0.0362) and laryngeal cancer (R = −0.93; p = 0.0025; Figure 2E/F). However, the percentage of patients with prostate cancer entering palliative care correlated positively with the year of diagnosis (R = 0.86; p = 0.0137). No time-dependent changes were noted for cancer of the lung, cervix, uterus, rectum, bladder or non-Hodgkin lymphoma.

**Table 1:** **Number of patients undergoing radical or palliative radiotherapy during the analysed period, according to diagnosis**

	Radical									Palliative									Overall
	2005	2006	2007	2008	2009	2010	2011	2012	Total	2005	2006	2007	2008	2009	2010	2011	2012	Total	
Breast	380	405	470	485	542	591	575	418	3866	125	82	79	84	66	78	56	43	613	4479
Lung	99	72	58	108	150	164	159	127	937	226	187	176	185	204	215	179	109	1481	2418
Prostate	84	109	122	111	152	201	276	153	1208	23	20	24	29	31	52	33	27	239	1447
Uterus	182	185	188	185	160	156	139	98	1293	2	2	1	3	1	2	5	3	19	1312
Cervix	152	150	181	160	156	155	152	83	1189	16	11	7	9	12	5	11	5	76	1265
Rectum	114	105	133	125	137	147	156	103	1020	43	27	27	41	18	31	27	12	226	1246
Brain	71	67	59	75	75	86	53	35	521	35	31	30	32	15	19	15	5	182	703
Larynx	46	77	99	68	66	77	88	47	568	25	32	23	21	10	11	4	3	129	697
Bladder	6	17	13	14	9	17	17	9	102	15	25	20	23	26	17	14	8	148	250
non-Hodgkin lymphoma	22	15	11	34	40	35	32	11	200	6	4	4	9	8	7	4	0	42	242
Stomach	12	7	25	26	40	41	39	24	214	3	4	1	1	0	4	0	2	15	229
Tonsil	16	21	27	18	13	23	27	12	157	14	9	14	11	10	6	2	4	70	227
Myeloma	9	2	6	5	7	9	8	5	51	27	27	20	24	21	17	25	12	173	224
Kidney	17	4	7	6	8	7	3	1	53	19	25	20	20	19	18	31	12	164	217
Hodgkin	39	38	40	13	15	20	18	16	199	2	0	3	0	3	2	1	2	13	212
Skin (non-melanoma)	29	22	15	14	13	19	18	12	142	13	6	6	6	2	5	1	3	42	184
Tongue	14	11	15	19	16	23	14	5	117	20	7	11	10	2	5	6	1	62	179
Pharynx	16	14	19	14	23	13	15	11	125	17	7	9	5	5	0	2	2	47	172
Melanoma	5	2	2	0	7	13	11	9	49	22	7	9	14	11	8	18	4	93	142
Testis	17	12	22	14	24	20	13	8	130	4	1	1	1	0	1	0	0	8	138
Thyroid gland	5	10	3	6	1	6	7	3	41	5	8	5	6	1	0	4	2	31	72
Soft tissue	1	2	3	8	13	11	11	8	57	1	2	1	1	3	4	1	0	13	70
Oral cavity	4	2	7	4	4	10	7	7	45	1	2	3	5	2	6	2	0	21	66
Oesophagus	6	3	2	3	2	3	6	6	31	2	5	7	1	4	3	4	3	29	60
Parotid gland	6	5	3	4	6	8	7	9	48	4	3	1	0	1	1	0	0	10	58
Ovary	2	8	5	1	3	1	3	1	24	4	3	1	6	5	4	7	1	31	55
Colon	0	2	0	0	2	1	1	4	10	6	5	4	5	4	7	3	2	36	46
Sinuses	2	3	3	3	5	5	6	2	29	3	1	1	1	2	0	1	0	9	38
Leukaemia	3	3	3	3	4	1	1	1	19	2	1	1	1	1	0	1	1	8	27
Other	56	55	48	48	48	71	81	64	471	31	27	24	24	19	15	29	11	180	651
Unlocalized	4	7	7	7	9	9	7	8	58	36	26	50	45	37	42	25	16	277	335
No Data	11	16	15	13	34	21	14	31	155	9	11	21	25	26	8	5	15	120	275
All diagnoses	1430	1451	1611	1594	1784	1964	1964	1331	13129	761	608	604	648	569	593	516	308	4607	17736

Data for 2012 is for 1st January– 30th June only.

**Figure 3:**
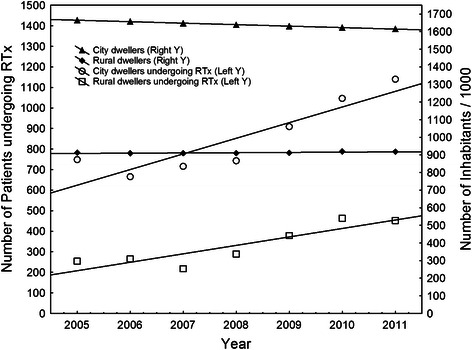
**The number of patients from urban and rural areas undergoing RTx (both palliative and radical) during the analysis period (left Y axis).** The right Y axis shows the number of inhabitants in rural or urban areas of the Lodz voivodeship administrative region, calculated using data of the Central Statistical Office.

Throughout the analysed period, the number of inhabitants in the region decreased from 2,577,465 in 2005 to 2,524,651 in 2012. A small but significant linear decrease in urbanization (percentage of patients living in towns or cities within the region) by a mean of 0.16% per year resulted in a drop from 64.61% in 2005 to 63.62% in 2012 (R = −0.98; p < 0.0001). Over the same period, the number of patients undergoing RTx, both radical and palliative, increased steadily, with a more pronounced rise noted amongst inhabitants of urban areas (Figure 3). This suggested increasing disparity in the likelihood of receiving RTx depending on the patient’s area of residence, with rural areas becoming progressively more neglected.

**Figure 4:**
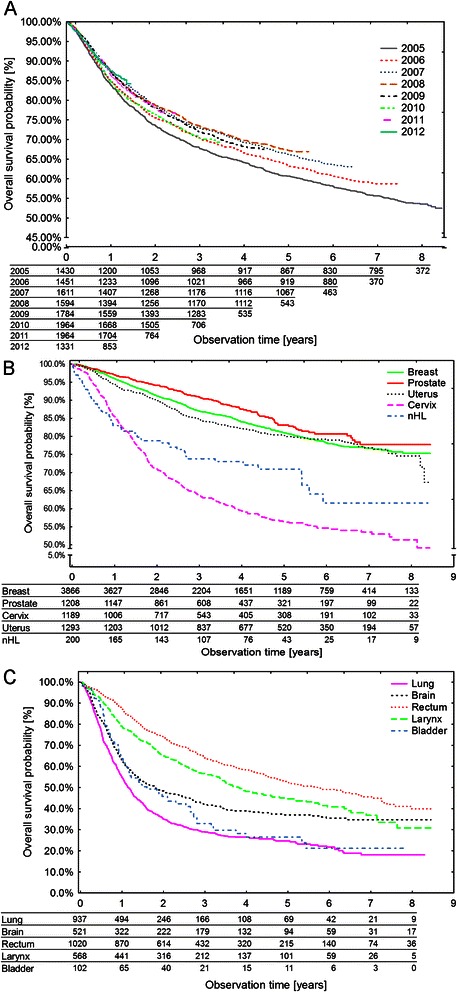
**Kaplan-Meier survival curves depicting changes of overall survival in the studied group, depending on the year of radiotherapy initiation (A); Kaplan-Meier survival curves for patients undergoing radical RTx for breast, prostate, cervical, or uterine cancer and non-Hodgkin lymphoma – five of the ten most frequent diagnoses with the best overall survival (B), and for lung, brain, laryngeal, bladder and rectal cancer – five of the ten most frequent diagnoses with the poorest overall survival (C).**

The probability of survival increased with the year of RTx initiation (Figure 4A), and the difference was statistically significant for a linear trend in RTx initiation year (HR = 0.97; 95% CI 0.96-0.99; p = 0.0013). Predictably, the diagnosis associated with the worst prognosis was lung cancer, with 26.26% of patients reaching 5-year survival. The best prognoses were associated with prostate and breast cancers (Figure 4B and C; Table 2).

**Table 2:** **Median Overall Survival (OS) times and 5-year OS rates for patients undergoing radical or palliative radiotherapy in the centre during the analysed period**

	Radical		Palliative		Whole group	
Diagnosis	Median OS	5Y OS rate	Median OS	5Y OS rate	Median OS	5Y OS rate
Breast	3.46	81.18%	1.11	19.11%	3.12	72.26%
Lung	1.05	24.54%	0.33	7.19%	0.55	13.80%
Prostate	3.02	83.32%	1.12	23.61%	2.77	73.33%
Uterus	4.26	80.14%	0.96	16.47%	4.18	79.40%
Cervix	2.71	56.35%	2.54	16.00%	0.77	54.21%
Brain	1.50	36.74%	0.50	10.62%	1.10	29.92%
Rectum	2.56	52.64%	0.78	6.55%	2.16	43.72%
Larynx	2.30	44.93%	1.90	10.95%	0.59	38.35%
Bladder	1.39	25.22%	0.36	9.31%	0.74	16.36%
non-Hodgkin lymphoma	3.26	70.65%	0.44	19.44%	2.73	62.97%
All	2.83	64.80%	0.52	12.87%	2.90	51.03%

The last row includes all patients from the dataset, including the top ten diagnoses listed within the table.

To verify that the impact of time on the improvement of OS rates was not solely due to the changing distribution of diagnoses, a multivariate proportional hazard regression model was developed to adjust for age, type of diagnosis (p < 0.0001) and year of RTx initiation. Results of the model confirmed the time-dependent improvement of prognosis, which was marginally greater in magnitude than in a univariate analysis (HR 0.96 95% CI 0.94-0.98; p < 0.0001), whereas older age at diagnosis was a detrimental factor (HR 1.03 95% CI 1.02-1.03; Additional file 1: Table S3).

## Discussion

The presented results provide reference material for epidemiological comparisons of RTx outcomes. Despite limited clinical data on tumour staging, the summarised results showed multiple interesting features relating to RTx availability, outcomes, and increasing urban/rural disparity. With regards to RTx availability, we noted that in cases of prostate cancer, the percentage of patients entering RTx with intention to treat in 2006 was equal to that in the United Kingdom [9,10], but then more than doubled during the analysis period. British estimates using the updated model published by Delaney et al. suggested that 48.3% of patients diagnosed with cancer will have an indication to radiotherapy [11], which seems to hold true in Poland as well.

An inherent problem associated with the analysis of patients undergoing RTx from a centre-based perspective is the lack of data on the actual diagnosis time. This ‘grey zone’ is caused by a distributed network of hospitals and clinics responsible for the diagnostic process and their own waiting times and delays. This could contribute to a spuriously shorter median survival and poorer OS, which may be of particular concern in cases of rapidly growing, aggressive tumours such as glioblastoma [12] or lung cancer [13]. Such delays are notoriously common, depend on cancer stage [14], and may have clinical consequences in the form of reduced odds of survival or a missed window of opportunity for radical treatment, as suggested in the study by Wai et al. [13]. However, in contrast to a British study showing a constantly increasing waiting time for breast cancer RTx [15], in our centre, the number of patients in RTx increased proportionally with the number of newly diagnosed cancer cases, suggesting that the availability of RTx did not decline. This may have been a result of shorter waiting times or allocation of more resources (Additional file 1: Figure S1). According to Polish National Health Fund (NFZ) the mean waiting time for radiotherapy start in October 2014 in Lodzkie Voivodeship was 39 days [16]. Moreover, the changing structure of radically and palliatively treated patients suggested that the delay between histopathological diagnosis and RTx could in fact decrease, allowing for more individuals to enter radical RTx, although this requires further, diagnosis-specific analyses. Another explanation of this shift would be the availability of more refined techniques which impact the decision making process and make more patients to be treated radically. The percentage of patients who required palliative treatment initially was far greater than estimated for Australian cohort [17]. It is probably explainable by higher number of patients diagnosed in later stages of diseases in Central Europe.

A crucial issue in developing the RTx survival database was that of coverage provided by the respective registries. To minimise the number of confounding factors, we relied on administrative resources. This had its downsides such as the lack of individual causes of death, which made it impossible for us to assess cause-specific survival. Nevertheless, the majority of our cohort comprised patients with either breast or prostate cancer, both of which are characterised by a wide-ranging percentage of cancer-attributable causes of death [18,19], making OS a far more robust and reliable parameter than cause-specific survival when no detailed clinical data are available.

Due to the database’s gradual development we were unable to ascertain patient socioeconomic status in 47.4% of cases. This may pose problems for long-term comparative analyses, as other registries such as the Eindhoven Cancer Registry have been collecting socioeconomic data since their inception [20] and shown socioeconomic status to be a major modifier of survival likelihood. The issue of socioeconomic status raises concerns about the apparent decline of treatment availability for patients in rural areas, which may lead to significant disparities in screening availability and treatment outcomes [21]. We identified no clear socioeconomic, administrative or health-related causes that would restrict access to this group of patients making it more likely, that the decreased proportion of patients from rural areas is due to insufficient resources and an increasing number of patients from urban areas entering treatment. This could result in patients migrating to or from the region, limiting the accessibility to available resources from the region’s inhabitants. We had no way of determining the true rate of migration between the Lodzkie Region and the adjacent Voivodeships. However, as we were able to discern that amongst the 9322 patients with available address data the fraction of immigrants from other regions was lower than 0.5%. It may be possible that the development of roads and better means of transportation increased patient mobility between the regions or improved access for patients from rural areas, but this remains speculative since no comparative, time-specific comparative groups are available to discern the impact of these factors in Poland. Finally it should be noted that distance and travel costs may obviously not be the only limiting factors restricting the access to medical care for patients with lower socioeconomic status.

The 5-year OS of patients with prostate cancer undergoing RTx was 73.3%. The overall relative survival estimate for the Polish centres participating in EUROCARE was of 70.7% [22]. In our group, no measures of net survival were possible as we did not possess information about the cause of death, nor total population mortality stratified by age and sex. Nevertheless, the survival rates suggested that our population did not differ much from that reported in the EUROCARE cohort. Given how the improvement in survival rate correlates with the year of diagnosis, the differences may be attributed to the delay in implementation of EUROCARE-4 survival reports and short-term observation of our patients, as the EUROCARE-4 cohort was recruited in the 1996–2002 period. German survival estimates from the Robert Koch Institute calculated for the year 2004 however, showed similar values to those of EUROCARE-4 [23], leading us to surmise that our current treatments provide our patients with probabilities of survival very similar to those in other western European countries.

The surprisingly good survival of patients with lung cancer may, however, be associated with a specific substructure of histopathological types noted in patients entering treatment, notably an underrepresentation of small-cell lung cancers. Other jurisdictions did not show much progress in improving the prognosis of individuals with lung cancer, with lower scores occurring in cohorts with greater representation of this kind of lung cancer [24,25]. Similar issues complicated a uniform interpretation of brain tumour therapeutic outcomes, as the registries generally do not include ICD-10 subcategory codes. Further integration of the presented dataset with histopathology and radiological databases will provide more opportunities for in-depth analyses of specific clinical factors.

In general, time-dependent improvement of OS rates may result from multiple factors, the most obvious of which is a changing structure of patients undergoing RTx. To verify that we evaluated the analysed cohort in terms of its structure (Additional file 1: Table S1) and patients’ age (Additional file 1: Table S2). We noted that even despite older patients entering the cohort in the later years of the analysis, the trend towards better survival was maintained (Additional file 1: Table S3). Thus, although in depth analyses for diagnosis-specific groups will be necessary to confirm whether the improvement is evident in all cancer types, overall it did seem that within our region a gradual improvement of OS noted over the last decade is a fact rather than a chance observation.

As the purpose of our study was to present the database with overall characteristics and outcomes of patients treated within the centre, we could not as this stage perform in-depth analyses of clinical and cancer-specific factors affecting survival. Further analyses will focus on cancer-specific trends and other factors after the database is merged with specific clinical datasets or the relevant data are extracted from patients’ medical records. The established framework of integrated cancer survival databases will allow for repeated follow-up analyses of the cohort in order to evaluate long-term survival.

## Conclusion

A minimal increase in the availability of RTx was noted in the region, with a noticeably growing disparity between urban and rural areas, the latter being under-resourced. Availability of RTx for Poland is increasing, although still below the optimal levels reported by other European academic RTx centres. Survival of patients undergoing radical RTx gradually improved throughout the analysed period, although it is still below the level achieved by leading RTx centres, potentially due to delayed diagnosis or other factors necessitating further in-depth analyses.

## Additional file

Additional file 1: **Table S1.** The numbers of patients that initiated radiotherapy during the analysed period. The ten most frequent diagnoses are enumerated separately, and show a marked increase in the numbers of patients with breast and prostate cancer referred to the RTx centre. The number of patients for 2012 covers the period January-June 2012 which is why no percentages of the National Cancer Registry (NCR) data are presented. Percentages in the third column represent the fraction of patients with newly diagnosed cancer who underwent radiotherapy. Percentages in columns for the ten most frequent diagnoses represent the fraction of all patients undergoing radiotherapy in a particular year. **Table S2.** Age of patients entering radiotherapy throughout the analysis period. Statistically significant trends of increasing age of patients were noted amongst breast, lung, prostate, uterine corpus, brain, rectum and bladder cancers (R coefficients and p values within main text). **Table S3.** Multivariate survival analysis results – lung cancer was chosen as the reference class for the “Diagnosis” categorical variable. Year was modelled in as a continuous variable which was consistent with the observed pattern of survival improvement. Models with year of diagnosis treated as a categorical variable produces a similar result (data not shown). HR – Hazard ratio, 95% CI – 95% confidence interval. **Figure S1.** Milestones of centre development, number of treated patients per year and equipment purchases. The blue bar represents the analysis period. **Figure S2.** Distribution of the top 10 diagnoses amongst patients undergoing radical (2a) and palliative RTx (2b).
