# Supplementary material for: Availability and outcomes of radiotherapy in Central Poland during the 2005-2012 period - an observational study
Source: BMC Cancer. 2015 Apr 2;15:214. doi: 10.1186/s12885-015-1236-7 (PMC4389344; doi:10.1186/s12885-015-1236-7)

Table S1 – The numbers of patients that initiated radiotherapy during the analysed period. The ten most frequent diagnoses are enumerated separately, and show a marked increase in the numbers of patients with breast and prostate cancer referred to the RTx centre. The number of patients for 2012 covers the period January-June 2012 which is why no percentages of the National Cancer Registry (NCR) data are presented. Percentages in the third column represent the fraction of patients with newly diagnosed cancer who underwent radiotherapy. Percentages in columns for the ten most frequent diagnoses represent the fraction of all patients undergoing radiotherapy in a particular year.

| Year of radiotherapy initiation | Patients diagnosed with cancer in the Lodz region according to the NCR | Number and percentage of patients undergoing radiotherapy | Breast | Lung | Prostate | Uterus | Cervix | Brain | Rectum | Larynx | Bladder | Non-Hodgkin lymphoma |
| --- | --- | --- | --- | --- | --- | --- | --- | --- | --- | --- | --- | --- |
| 2005 | 9307 | 2191 (23.71%) | 505 (23.05%) | 325 (14.83%) | 107 (4.88%) | 184 (8.40%) | 168 (7.67%) | 106 (4.84%) | 157 (7.17%) | 71 (3.24%) | 21 (0.96%) | 28 (1.28%) |
| 2006 | 9068 | 2059 (22.82%) | 487 (23.65%) | 259 (12.58%) | 129 (6.27%) | 187 (9.08%) | 161 (7.82%) | 98 (4.76%) | 132 (6.41%) | 109 (5.29%) | 42 (2.04%) | 19 (0.92%) |
| 2007 | 8516 | 2215 (26.08%) | 549 (24.79%) | 234 (10.56%) | 146 (6.59%) | 189 (8.53%) | 188 (8.49%) | 89 (4.02%) | 160 (7.22%) | 122 (5.51%) | 33 (1.49%) | 15 (0.68%) |
| 2008 | 9218 | 2242 (24.35%) | 569 (25.38%) | 293 (13.07%) | 140 (6.24%) | 188 (8.39%) | 169 (7.54%) | 107 (4.77%) | 166 (7.40%) | 89 (3.97%) | 37 (1.65%) | 43 (1.92%) |
| 2009 | 9860 | 2353 (23.87%) | 608 (25.84%) | 354 (15.04%) | 183 (7.78%) | 161 (6.84%) | 168 (7.14%) | 90 (3.82%) | 155 (6.59%) | 76 (3.23%) | 35 (1.49%) | 48 (2.04%) |
| 2010 | 10199 | 2557 (25.10%) | 669 (26.16%) | 379 (14.82%) | 253 (9.89%) | 158 (6.18%) | 160 (6.26%) | 105 (4.11%) | 178 (6.96%) | 88 (3.44%) | 34 (1.33%) | 42 (1.64%) |
| 2011 | 10221 | 2480 (24.26%) | 631 (25.44%) | 338 (13.63%) | 309 (12.46%) | 144 (5.81%) | 163 (6.57%) | 68 (2.74%) | 183 (7.38%) | 92 (3.71%) | 31 (1.25%) | 36 (1.45%) |
| 2012 | 11088 | 1636 | 459 | 236 | 180 | 99 | 88 | 40 | 115 | 50 | 17 | 11 |

Table S2 – Age of patients entering radiotherapy throughout the analysis period. Statistically significant trends of increasing age of patients were noted amongst breast, lung, prostate, uterine corpus, brain, rectum and bladder cancers (R coefficients and p values within main text).

| Diagnosis | Year of diagnosis | Median (25-75%) [years] | Mean age+/-Std. Dev. [years] | Minimum [years] | Maximum [years] | N |
| --- | --- | --- | --- | --- | --- | --- |
| Breast | 2005 | 55.4 (49.2-62.1) | 56.2+/-10.1 | 26.30 | 85.40 | 508 |
| Breast | 2006 | 56.9 (49.9-64.7) | 56.8+/-10.8 | 28.19 | 79.59 | 488 |
| Breast | 2007 | 55.9 (49.9-63.8) | 56.6+/-10.5 | 26.79 | 91.67 | 550 |
| Breast | 2008 | 57.0 (50.3-62.5) | 56.8+/-9.59 | 31.15 | 85.11 | 570 |
| Breast | 2009 | 57.6 (50.7-63.0) | 57.1+/-10.3 | 30.21 | 85.01 | 608 |
| Breast | 2010 | 57.4 (51.4-63.3) | 57.3+/-9.98 | 29.98 | 95.94 | 669 |
| Breast | 2011 | 58.4 (51.2-64.1) | 57.5+/-10.5 | 23.45 | 87.00 | 631 |
| Breast | 2012 | 60.1 (52.1-65.2) | 59.0+/-10.6 | 25.56 | 83.12 | 462 |
| Lung | 2005 | 59.7 (54.3-67.5) | 60.8+/-9.23 | 33.53 | 84.82 | 327 |
| Lung | 2006 | 59.8 (55.0-67.2) | 60.9+/-8.77 | 29.82 | 85.00 | 260 |
| Lung | 2007 | 60.8 (55.0-68.2) | 61.6+/-8.48 | 40.31 | 81.86 | 235 |
| Lung | 2008 | 60.7 (56.2-67.4) | 61.4+/-8.53 | 37.13 | 87.47 | 293 |
| Lung | 2009 | 61.6 (56.2-66.8) | 61.7+/-8.03 | 41.51 | 82.66 | 354 |
| Lung | 2010 | 62.2 (57.0-68.7) | 62.5+/-8.38 | 25.93 | 85.67 | 379 |
| Lung | 2011 | 62.4 (57.0-68.0) | 62.8+/-8.30 | 39.52 | 83.37 | 338 |
| Lung | 2012 | 63.2 (57.9-68.2) | 63.4+/-7.68 | 42.77 | 79.19 | 236 |
| Prostate | 2005 | 69.6 (61.5-73.1) | 67.7+/-7.46 | 52.15 | 82.07 | 107 |
| Prostate | 2006 | 68.5 (63.3-72.4) | 67.7+/-6.78 | 47.55 | 83.07 | 129 |
| Prostate | 2007 | 67.1 (61.8-72.6) | 68.2+/-7.03 | 46.61 | 87.50 | 146 |
| Prostate | 2008 | 66.3 (61.9-72.0) | 66.7+/-7.13 | 49.72 | 85.63 | 140 |
| Prostate | 2009 | 68.3 (62.6-73.8) | 67.8+/-7.33 | 42.39 | 84.41 | 183 |
| Prostate | 2010 | 66.8 (62.4-71.9) | 67.0+/-7.45 | 49.39 | 86.72 | 254 |
| Prostate | 2011 | 67.9 (63.2-73.7) | 68.1+/-7.07 | 48.97 | 84.14 | 309 |
| Prostate | 2012 | 68.6 (63.4-74.3) | 68.6+/-7.07 | 52.66 | 86.98 | 180 |
| Uterus | 2005 | 61.5 (55.6-68.9) | 61.8+/-9.32 | 23.39 | 82.10 | 186 |
| Uterus | 2006 | 63.7 (56.0-69.6) | 62.7+/-8.60 | 38.49 | 80.13 | 189 |
| Uterus | 2007 | 60.7 (56.0-69.1) | 62.1+/-8.92 | 35.66 | 85.07 | 189 |
| Uterus | 2008 | 63.7 (56.6-69.5) | 62.9+/-9.47 | 36.70 | 86.13 | 188 |
| Uterus | 2009 | 62.5 (57.0-68.4) | 62.3+/-8.55 | 28.27 | 84.29 | 161 |
| Uterus | 2010 | 64.1 (56.9-71.7) | 63.6+/-10.4 | 31.36 | 83.41 | 158 |
| Uterus | 2011 | 62.4 (57.0-70.8) | 63.2+/-9.14 | 40.00 | 88.65 | 145 |
| Uterus | 2012 | 61.5 (56.7-67.3) | 62.5+/-7.69 | 39.27 | 78.07 | 101 |
| Cervix | 2005 | 56.8 (48.1-63.5) | 55.9+/-12.3 | 23.88 | 85.25 | 169 |
| Cervix | 2006 | 57.1 (49.2-65.8) | 57.5+/-11.7 | 27.01 | 85.90 | 162 |
| Cervix | 2007 | 56.3 (48.6-63.5) | 56.6+/-11.8 | 27.62 | 84.52 | 188 |
| Cervix | 2008 | 56.8 (50.0-65.9) | 58.1+/-12.2 | 28.48 | 97.39 | 169 |
| Cervix | 2009 | 57.9 (50.4-68.2) | 58.8+/-11.5 | 26.89 | 85.98 | 168 |
| Cervix | 2010 | 58.5 (52.5-66.4) | 59.2+/-11.5 | 29.24 | 89.78 | 161 |
| Cervix | 2011 | 58.0 (50.3-64.2) | 57.9+/-12.2 | 27.48 | 88.75 | 163 |
| Cervix | 2012 | 57.1 (46.6-63.3) | 57.0+/-12.9 | 32.31 | 95.46 | 88 |
| Brain | 2005 | 52.8 (38.8-62.5) | 50.9+/-15.5 | 18.95 | 79.69 | 108 |
| Brain | 2006 | 52.1 (45.6-59.6) | 51.4+/-12.8 | 18.48 | 77.33 | 98 |
| Brain | 2007 | 55.7 (44.5-64.5) | 54.7+/-13.2 | 24.89 | 81.00 | 89 |
| Brain | 2008 | 54.1 (45.4-61.9) | 53.3+/-14.4 | 19.08 | 82.98 | 107 |
| Brain | 2009 | 56.6 (50.9-65.2) | 56.1+/-13.0 | 20.37 | 88.93 | 90 |
| Brain | 2010 | 58.5 (50.5-64.5) | 56.1+/-14.0 | 19.28 | 80.69 | 105 |
| Brain | 2011 | 58.6 (48.9-67.8) | 56.7+/-13.7 | 20.04 | 79.65 | 68 |
| Brain | 2012 | 57.3 (43.3-64.9) | 54.3+/-13.3 | 27.07 | 80.54 | 40 |
| Rectum | 2005 | 61.9 (54.2-69.9) | 61.9+/-10.1 | 38.32 | 82.39 | 157 |
| Rectum | 2006 | 62.7 (55.4-69.3) | 61.8+/-10.6 | 27.60 | 85.96 | 133 |
| Rectum | 2007 | 63.2 (56.6-71.2) | 63.4+/-10.1 | 28.98 | 88.29 | 160 |
| Rectum | 2008 | 63.4 (56.9-71.3) | 63.6+/-10.0 | 33.02 | 91.52 | 166 |
| Rectum | 2009 | 63.4 (55.9-70.3) | 63.3+/-10.0 | 31.21 | 85.53 | 155 |
| Rectum | 2010 | 63.2 (56.5-71.5) | 63.5+/-10.3 | 25.60 | 92.15 | 179 |
| Rectum | 2011 | 65.3 (58.6-73.5) | 65.5+/-9.79 | 32.58 | 84.25 | 183 |
| Rectum | 2012 | 66.9 (61.4-73.0) | 66.9+/-9.52 | 26.81 | 88.12 | 115 |
| Larynx | 2005 | 57.8 (53.1-66.5) | 59.6+/-10.4 | 28.76 | 85.05 | 71 |
| Larynx | 2006 | 62.2 (56.0-70.2) | 63.4+/-10.3 | 42.80 | 92.81 | 109 |
| Larynx | 2007 | 60.7 (54.2-68.3) | 61.6+/-9.04 | 47.18 | 86.03 | 123 |
| Larynx | 2008 | 62.1 (56.5-68.9) | 62.5+/-8.43 | 45.22 | 82.41 | 89 |
| Larynx | 2009 | 59.8 (56.7-65.8) | 61.5+/-8.33 | 45.32 | 87.22 | 76 |
| Larynx | 2010 | 61.5 (56.3-67.6) | 62.0+/-8.97 | 39.01 | 82.15 | 88 |
| Larynx | 2011 | 63.4 (58.9-68.9) | 63.7+/-8.98 | 27.26 | 84.82 | 92 |
| Larynx | 2012 | 61.1 (57.2-67.1) | 61.9+/-7.24 | 47.30 | 79.21 | 50 |
| Bladder | 2005 | 65.2 (54.1-75.9) | 64.2+/-12.2 | 45.52 | 81.37 | 21 |
| Bladder | 2006 | 67.6 (60.1-73.9) | 66.4+/-10.5 | 29.16 | 84.23 | 43 |
| Bladder | 2007 | 65.2 (56.4-71.4) | 63.5+/-9.03 | 45.21 | 76.05 | 33 |
| Bladder | 2008 | 70.6 (62.2-76.7) | 67.5+/-12.3 | 28.10 | 86.90 | 38 |
| Bladder | 2009 | 65.3 (61.4-71.4) | 66.1+/-8.24 | 50.42 | 85.58 | 35 |
| Bladder | 2010 | 64.9 (59.9-73.7) | 66.1+/-9.63 | 45.45 | 88.03 | 34 |
| Bladder | 2011 | 68.1 (56.4-76.2) | 66.5+/-13.0 | 30.57 | 87.70 | 31 |
| Bladder | 2012 | 67.6 (58.8-76.1) | 67.5+/-10.1 | 51.02 | 82.70 | 17 |
| non-Hodgkin lymphoma | 2005 | 64.0 (50.6-70.6) | 57.9+/-17.9 | 22.67 | 80.53 | 28 |
| non-Hodgkin lymphoma | 2006 | 56.7 (53.3-72.1) | 62.0+/-15.8 | 27.93 | 86.65 | 19 |
| non-Hodgkin lymphoma | 2007 | 50.9 (28.6-69.6) | 49.7+/-19.3 | 21.95 | 74.63 | 15 |
| non-Hodgkin lymphoma | 2008 | 52.5 (38.4-60.4) | 50.8+/-18.6 | 20.84 | 91.16 | 43 |
| non-Hodgkin lymphoma | 2009 | 43.8 (28.4-67.3) | 48.8+/-22.2 | 18.62 | 91.04 | 49 |
| non-Hodgkin lymphoma | 2010 | 57.4 (32.4-69.9) | 52.5+/-19.8 | 19.24 | 84.10 | 42 |
| non-Hodgkin lymphoma | 2011 | 62.6 (37.2-74.6) | 57.7+/-19.7 | 25.16 | 86.78 | 36 |
| non-Hodgkin lymphoma | 2012 | 60.1 (47.0-77.0) | 59.7+/-14.7 | 36.16 | 80.79 | 11 |
| Overall | 2005 | 58.5 (51.6-67.7) | 58.7+/-12.1 | 18.07 | 91.75 | 2207 |
| Overall | 2006 | 59.7 (52.4-68.4) | 59.6+/-11.8 | 17.98 | 92.81 | 2069 |
| Overall | 2007 | 59.2 (52.1-67.2) | 59.4+/-11.5 | 18.85 | 91.67 | 2221 |
| Overall | 2008 | 59.7 (52.8-67.3) | 59.5+/-11.5 | 17.99 | 97.39 | 2245 |
| Overall | 2009 | 60.2 (53.5-67.6) | 59.9+/-11.4 | 18.02 | 91.04 | 2354 |
| Overall | 2010 | 61.2 (54.1-68.2) | 60.5+/-11.3 | 18.02 | 95.94 | 2560 |
| Overall | 2011 | 62.0 (55.4-68.8) | 61.4+/-11.3 | 20.04 | 89.62 | 2481 |
| Overall | 2012 | 61.9 (55.6-69.3) | 61.7+/-11.3 | 19.71 | 95.46 | 1642 |

Table S3 – Multivariate survival analysis results – lung cancer was chosen as the reference class for the “Diagnosis” categorical variable. Year was modelled in as a continuous variable which was consistent with the observed pattern of survival improvement. Models with year of diagnosis treated as a categorical variable produces a similar result (data not shown). HR – Hazard ratio, 95%CI – 95% confidence interval,

| Variable | Estimate | HR | p |
| --- | --- | --- | --- |
| Year of diagnosis | -0.04 | 0.96 (0.95-0.98) | 0.0000 |
| Age at diagnosis | 0.03 | 1.03 (1.02-1.03) | 0.0000 |
| **Diagnosis type** |  |  |  |
| Breast | -0.90 | 0.05 (0.04-0.06) | 0.0000 |
| Prostate | -1.26 | 0.01 (0.01-0.02) | 0.0000 |
| Uterus | -0.99 | 0.02 (0.01-0.02) | 0.0000 |
| Cervix | 0.07 | 0.13 (0.1-0.17) | 0.1191 |
| Brain | 0.96 | 0.33 (0.24-0.44) | 0.0000 |
| Rectum | 0.04 | 0.18 (0.13-0.24) | 0.3587 |
| Larynx | 0.33 | 0.54 (0.41-0.7) | 0.0000 |
| Bladder | 0.80 | 0.74 (0.58-0.93) | 0.0000 |
| non-Hodgkin lymphoma | -0.15 | 0.28 (0.22-0.36) | 0.1702 |

Figure S1 - Milestones of centre development, number of treated patients per year and equipment purchases. The blue bar represents the analysis period.


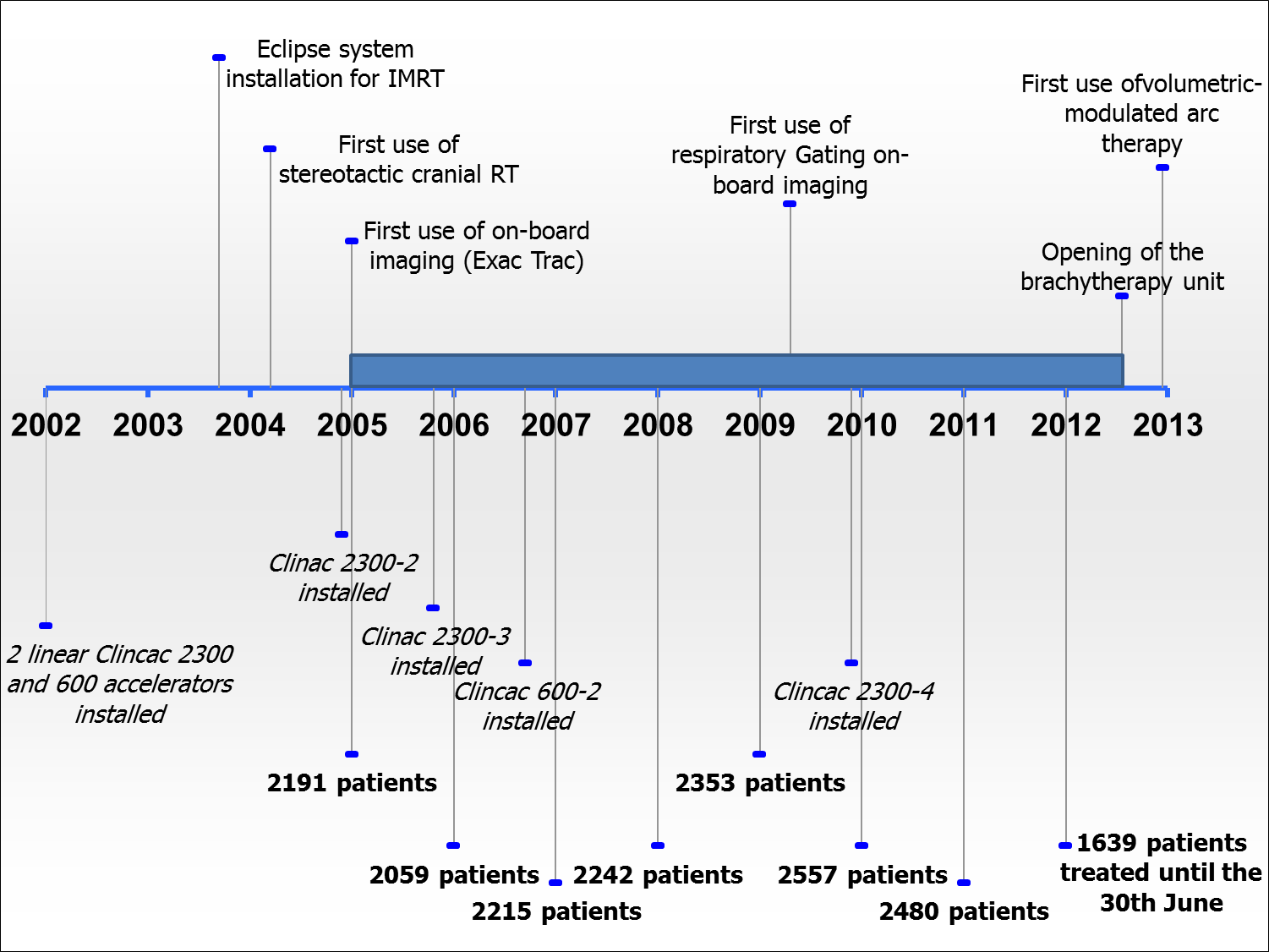


Figure S2 - Distribution of the top 10 diagnoses amongst patients undergoing radical (2a) and palliative RTx (2b).


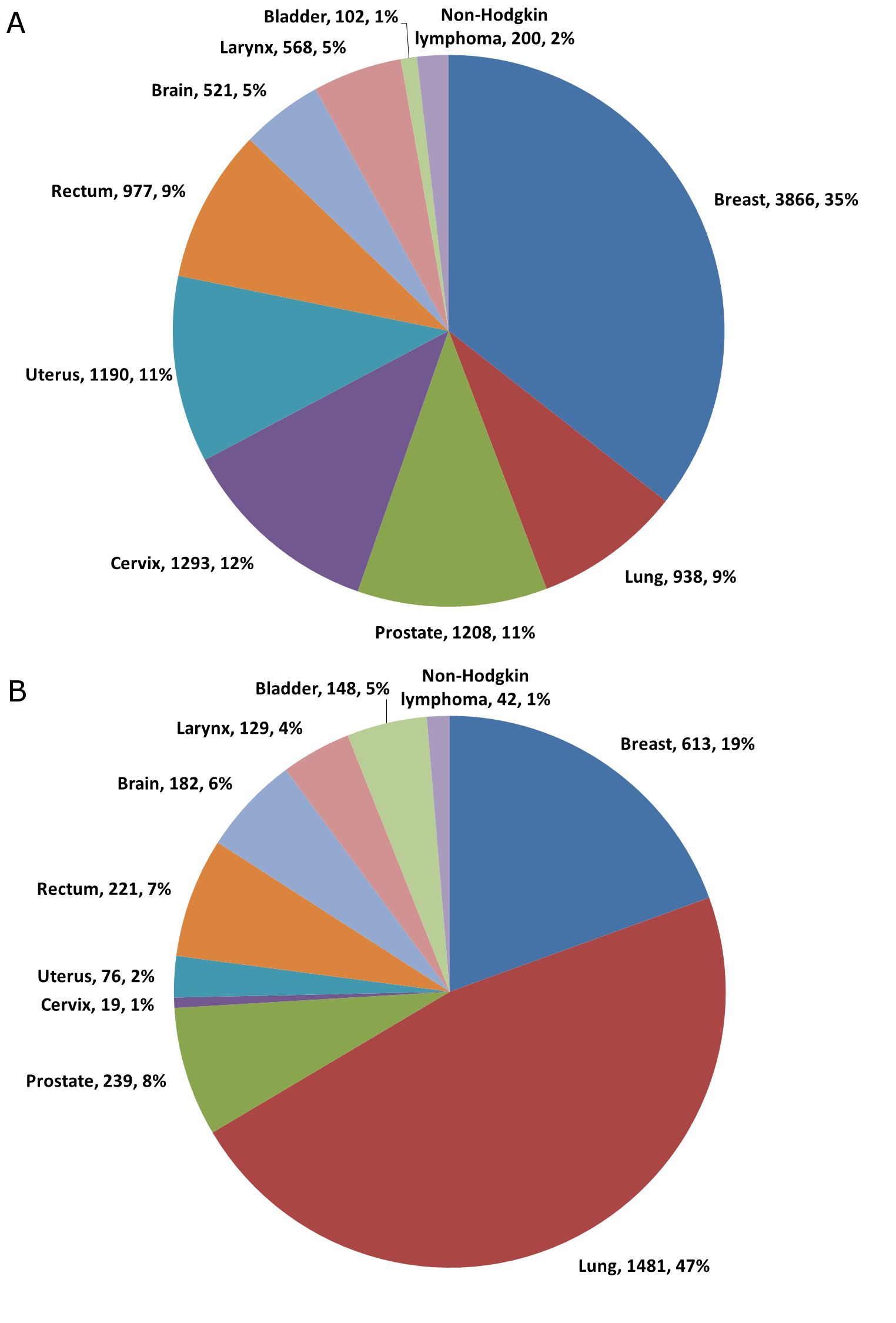

Supplement: Additional file 1: — Table S1. The numbers of patients that initiated radiotherapy during the analysed period. The ten most frequent diagnoses are enumerated separately, and show a marked increase in the numbers of patients with breast and prostate cancer referred to the RTx centre. The number of patients for 2012 covers the period January-June 2012 which is why no percentages of the National Cancer Registry (NCR) data are presented. Percentages in the third column represent the fraction of patients with newly diagnosed cancer who underwent radiotherapy. Percentages in columns for the ten most frequent diagnoses represent the fraction of all patients undergoing radiotherapy in a particular year. Table S2. Age of patients entering radiotherapy throughout the analysis period. Statistically significant trends of increasing age of patients were noted amongst breast, lung, prostate, uterine corpus, brain, rectum and bladder cancers (R coefficients and p values within main text). Table S3. Multivariate survival analysis results – lung cancer was chosen as the reference class for the “Diagnosis” categorical variable. Year was modelled in as a continuous variable which was consistent with the observed pattern of survival improvement. Models with year of diagnosis treated as a categorical variable produces a similar result (data not shown). HR – Hazard ratio, 95% CI – 95% confidence interval. Figure S1. Milestones of centre development, number of treated patients per year and equipment purchases. The blue bar represents the analysis period. Figure S2. Distribution of the top 10 diagnoses amongst patients undergoing radical (2a) and palliative RTx (2b). [file 12885_2015_1236_MOESM1_ESM.docx]
